# Assessing breast density using the chemical-shift encoding-based proton density fat fraction in 3-T MRI

**DOI:** 10.1007/s00330-022-09341-x

**Published:** 2022-12-20

**Authors:** Tabea Borde, Mingming Wu, Stefan Ruschke, Christof Boehm, Jonathan Stelter, Kilian Weiss, Stephan Metz, Marcus Richard Makowski, Dimitrios C. Karampinos, Eva Maria Fallenberg

**Affiliations:** 1 Department of Diagnostic and Interventional Radiology, Klinikum rechts der Isar, TUM School of Medicine, Technical University of Munich, Ismaninger Str. 22, 81675 Munich, Germany; 2 Philips GmbH, Hamburg, Germany

**Keywords:** Breast, Breast density, Magnetic resonance imaging

## Abstract

**Objectives:**

There is a clinical need for a non-ionizing, quantitative assessment of breast density, as one of the strongest independent risk factors for breast cancer. This study aims to establish proton density fat fraction (PDFF) as a quantitative biomarker for fat tissue concentration in breast MRI and correlate mean breast PDFF to mammography.

**Methods:**

In this retrospective study, 193 women were routinely subjected to 3-T MRI using a six-echo chemical shift encoding-based water-fat sequence. Water-fat separation was based on a signal model accounting for a single T_2_* decay and a pre-calibrated 7-peak fat spectrum resulting in volumetric fat-only, water-only images, PDFF- and T_2_*-values. After semi-automated breast segmentation, PDFF and T_2_* values were determined for the entire breast and fibroglandular tissue. The mammographic and MRI-based breast density was classified by visual estimation using the American College of Radiology Breast Imaging Reporting and Data System categories (ACR A-D).

**Results:**

The PDFF negatively correlated with mammographic and MRI breast density measurements (Spearman rho: −0.74, *p* < .001) and revealed a significant distinction between all four ACR categories. Mean T_2_* of the fibroglandular tissue correlated with increasing ACR categories (Spearman rho: 0.34, *p* < .001). The PDFF of the fibroglandular tissue showed a correlation with age (Pearson rho: 0.56, *p* = .03).

**Conclusion:**

The proposed breast PDFF as an automated tissue fat concentration measurement is comparable with mammographic breast density estimations. Therefore, it is a promising approach to an accurate, user-independent, and non-ionizing breast density assessment that could be easily incorporated into clinical routine breast MRI exams.

**Key Points:**

•* The proposed PDFF strongly negatively correlates with visually determined mammographic and MRI-based breast density estimations and therefore allows for an accurate, non-ionizing, and user-independent breast density measurement*.

• In combination with T2*, the PDFF can be used to track structural alterations in the composition of breast tissue for an individualized risk assessment for breast cancer.

## Introduction

Breast cancer accounts for almost 25% of all diagnosed cancers in women and is the leading cause of cancer-related deaths among women worldwide [1]. In both screen-detected and symptomatic patients, high breast density has been identified as one of the strongest independent risk factors and is associated with a twofold increase in breast cancer incidence rates [2–4].

In clinical practice, the breast density is assessed in mammograms by visual estimation of the content of radiopaque fibroglandular parenchyma within the breast and classified according to the American College of Radiology (ACR) categories under the Breast Imaging Reporting and Data System® (BI-RADS®, 5^th^ edition) in 4 grades: (A) almost entirely fatty, (B) scattered fibroglandular densities, (C) heterogeneously dense, which may obscure small masses, and (D) extremely dense, which lowers the sensitivity of mammography [5]. However, several limitations lead to a non-consistent intra- and inter-reader variability of mammography-based breast density measurements. First, the technical execution requires a compression of the breast which is then exposed to low-dose ionizing radiation. Slight deviations in tissue compression or radiation exposure calibration may already confound breast density measurements [6]. Furthermore, the resulting two-dimensional projection of the volumetric body has a limited capacity to provide objective proportions of the breast composition [4]. Altogether, these factors combined with the non-neglectable, detrimental exposure to ionizing radiation, especially at younger age, challenge the accuracy and reliability of mammography in assessing breast density and cancer risk prediction.

Magnetic resonance imaging (MRI) presents a suitable alternative for an objective, volumetric quantification of the structural composition of the breast without the use of ionizing radiation. Standard T_1_-weighted imaging techniques have been primarily used to separate adipose and fibroglandular breast tissue [7, 8]. Recent studies using chemical shift encoding-based water-fat separation (Dixon imaging) improved the contrast in separating fat from water proton signals in the breast. These approaches yielded favorable results in the differentiation of adipose and fibroglandular breast tissue and the measurement of a signal-weighted breast fat fraction but are dependent on the imaging protocol and the breast density segmentation processing pipeline [9–11]. A promising quantitative biomarker for the tissue fat concentration is the water-fat MRI-based proton density fat fraction (PDFF). The PDFF is defined as the ratio of density of triglyceride protons to the total density of triglyceride and water protons and therefore accurately reflects the concentration of fat within that tissue. The PDFF values derive directly from automatically calculated PDFF maps that are insensitive to changes in acquisition parameters which renders the PDFF a comprehensive and clinically practical biomarker [12–14]. In previous studies, the PDFF has been successfully correlated to different structural tissues such as muscle, pancreas, and liver [15–17] and has been only recently applied for breast density assessment [18, 19].

Therefore, the purpose of this study is to establish the proton density fat fraction as a quantitative biomarker for fat tissue concentration in breast MRI by correlating its outcome to mammography as the most widely used breast density measurement in clinical practice.

## Material and methods

### Study population

**Fig. 1:**
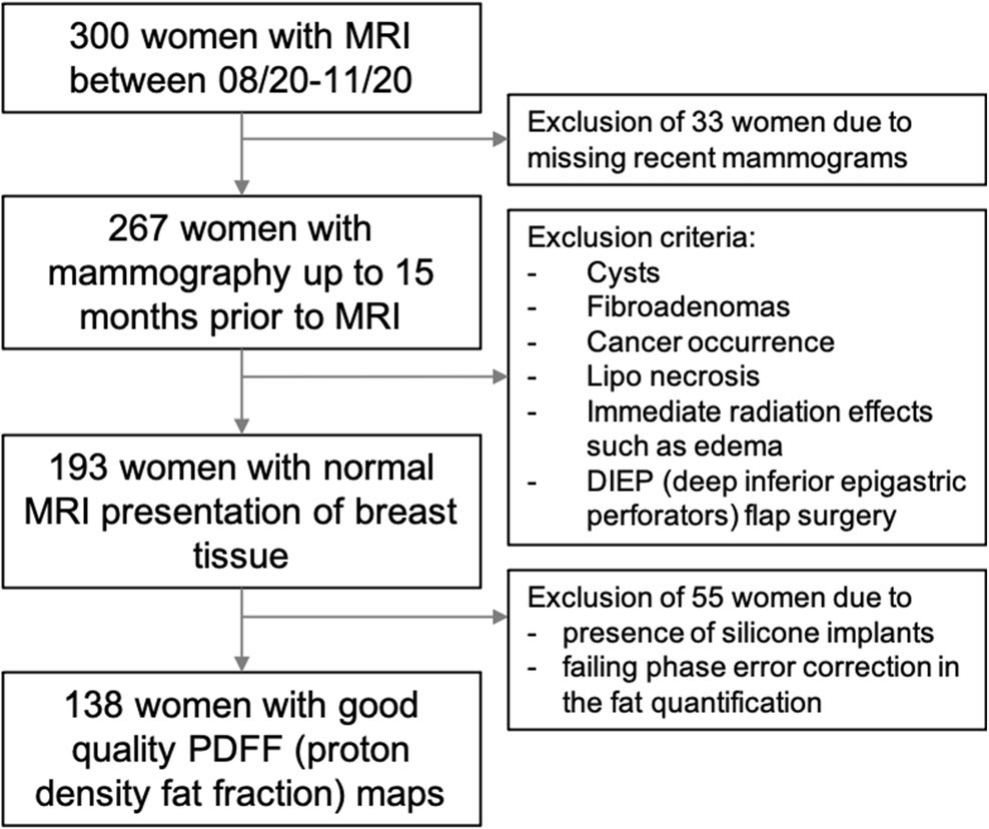
Study flow chart

This institutional review board–approved retrospective study initially included 300 pre- and postmenopausal women out of which 193 women (median age 48 years, range 29–81 years) had received digital mammography within 1–15 months (median 9) prior to the conductance of the MRI scan from August to November 2020 (Fig. 1). Indication for breast imaging was either screening in high-risk patients, surveillance of cancer patients, or further work up of unclear findings.

### Breast density measurements

Qualitative breast density measurements in two-dimensional mammograms, currently the most widely used breast density estimation in clinical practice, were used as the clinical standard reference. Mammography was performed by using full-field digital mammographic units (GE Healthcare). The visual evaluation of the composition of breast tissue was performed in the craniocaudal and mediolateral-oblique view and classified into the American College of Radiology (ACR) Breast Imaging Reporting and Data System® (BI-RADS®) categories, 5^th^ edition [20], by the reporting radiologist (4 years of experience in mammography) as well as an independent reader (1 year of experience in mammography) blinded to the study in one session. The inter-reader reliability was assessed using Cohen’s kappa.

Furthermore, the same visual evaluation was performed in MR T1-weighted images according to the ACR-BIRADS categories by the same reporting and independent radiologists blinded to the results of the mammographic readings.

### MR measurements

Each woman was examined in prone position using a dedicated 7-channel breast radiofrequency (RF) coil in a 3.0-T MRI scanner (Philips Ingenia, Philips Healthcare). In addition to conventional T_1_- and T_2_-weighted sequences, a six-echo 3D spoiled gradient-echo sequence employing bipolar imaging readouts in axial plane was routinely included with the following parameters: 1.7 mm isotropic acquisition voxel size, field of view (FOV, mm): AP = 220, RL = 440, FH = 190, TR/TE/ΔTE = 8.8/1.43/1.1, flip angle = 3°, scan time: 2 min 11 s per stack. A combination of sensitivity encoding (SENSE) and compressed sensing was employed based on the vendor’s implementation (Compressed SENSE, Philips Healthcare) with an acceleration factor equal to 3. This proton density Dixon sequence was part of the clinical routine protocol to provide artifact-free 3D fat-suppressed images before contrast injection.

**Fig. 2:**
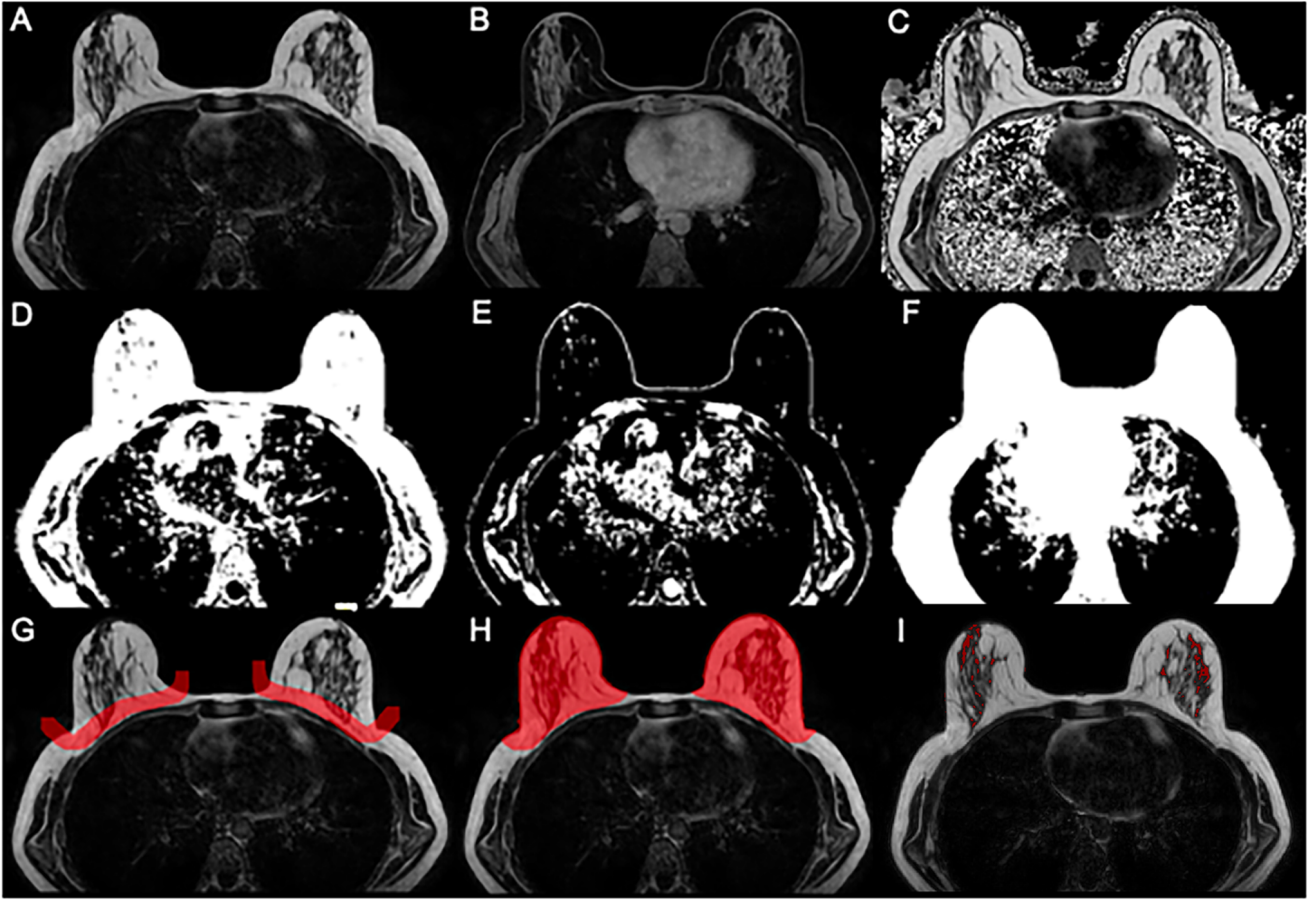
Segmentation workflow. **A** Axial MRI fat-separated image. **B** Axial MRI water-separated image. **C** PDFF map. **D**–**F** Fat, water, and foreground mask as a result of k-means clustering. **G** Manual segmentation delineating the border between the pectoralis muscle and breast parenchyma. **H** Automated completion of segmentation masks of left and right mask. **I** Final automated segmentation of the fibroglandular tissue

After phase error correction, water-fat separation was performed online using the water-fat separation algorithm of the vendor (mDixon Quant, Philips Healthcare). Water-fat separation was performed based on a signal model accounting for a single T_2_* decay and a pre-calibrated 7-peak fat spectrum accounting for the presence of multiple peaks in the fat spectrum. Resulting volumetric fat-only, water-only images, PDFF maps (Fig. 2), and T_2_* values were generated. The PDFF maps were automatically computed as the ratio of the fat signal over the sum of fat and water signals. The visual estimation of the breast density in conventional MR in native T1-weighted images according to the BI-RADS classification was performed by a radiologist with 10 years and a radiologist with 1 year of experience in MR breast readings blinded to the study in one session.

### MR image processing

Breast density analysis was performed on breasts unaffected by cancer, fibroadenomas, cysts, lipo-necrosis after treatment or immediate radiation effects, and without breast augmentation or implantation material to minimize interference with the breast density analysis. Semi-automated breast segmentation was performed by manually delineating the border between the pectoralis muscle and the breast parenchyma on every second slice in fat-only images using the open-source segmentation program ITK-Snap (www.itksnap.org, version 3.8.0, Fig. 2G) [21]. Using the Image processing toolbox (MATLAB R2020b, The MathWorks), k-means clustering was performed, and the FOV was divided into water, fat, and background signal. The manually selected borders were connected with a dilation step and the centroids of the left and right breast were calculated by projecting the manual delineation in AP orientation. Both the centroid position and the border delineation helped separating the breast from the thorax using “bwselect” from the Image processing toolbox. Finally, the mean PDFF over the entire breast and the mean PDFF and T_2_* over only the fibroglandular tissue of the isolated breasts were automatically extracted. The fibroglandular tissue was segmented using the sharp delineation between fat and fibroglandular tissue as well as a fat-concentration cut-off of 20% as fibroglandular tissue empirically contains very low fat signal (0–10%). All segmentation steps were performed by the same operator. The workflow of the image segmentation is illustrated in Fig. 2.

### Statistical analysis

All statistical analyses were performed using the open-source software R (R Foundation for Statistical Computing, version 1.3.1056, 2020). The Shapiro-Wilk normality test revealed a non-Gaussian distribution of the nominal variables including age, and the PDFF and T_2_* of the entire breast and the fibroglandular tissue. Calculations of the left and right breast were compared using the intraclass correlation coefficient (ICC) followed by paired *t*-tests. To reduce bias, one breast in each woman was chosen according to the exclusion criteria for further analysis (Fig. 1). The inter-reader reliability of the mammographic and MRI-based breast density estimations was assessed using Cohen’s kappa. The Cohen’s kappa reference values were used established by McHugh et al [22]. To test the concordance between the categorical mammographic and nominal MRI-based breast density measurements, the Spearman correlation coefficient *r* was calculated. To examine the relationship between the categorical, visually estimated mammographic and MRI-based breast density, the chi-squared test of independence was performed. The PDFF values were plotted in relation to the four ACR breast density categories. To evaluate their congruity, the Kruskal-Wallis test was performed followed by the Bonferroni-Holm correction criteria to adjust the *p* values in multiple comparisons due to their non-Gaussian distribution. To test for differences of average measures, paired *t*-tests were used. A two-tailed *p* value < 0.05 was considered statistically significant.

## Results

Evaluable PDFF maps were obtained in 138 women, excluding 55 patients due to different sources of artifacts. In detail, 21 datasets were excluded due to the presence of silicone implants (current Dixon processing does not account for silicone signals). In addition, 34 datasets were excluded due to a failing phase error correction in the fat quantification (Fig. 1). The failing phase error correction step resulted in erroneous fat fraction values over the entire field of view and was mostly likely related to an erroneous phase error estimation due to the lack of signal in the lungs within a large portion of the imaging field of view.

Patient and breast characteristics are displayed in Table 1. In this patient cohort, 91 women (66 %) presented with a reported history of breast cancer treated with surgical breast conserving therapy (*n* = 58), and/or a combination of radiation, chemotherapy, and additional antihormonal treatment (*n* = 46). All cancer patients revealed normal MRI follow-ups without any signs of recurrence. Separate calculations for right and left breasts showed similar results with a range of ICC of 0.997 to 0.999 (*p* < .001) for breast density and glandular tissue, and paired *t*-tests demonstrated no significant differences (*p =* .94). The chi-squared test revealed a significant relationship between visual mammographic and conventional MRI-based breast density readings (*p* < .001). The inter-reader reliability in mammographic breast density estimations was found to be low (*k* = 0.5, 95% confidence interval [CI] 0.38, 0.62) [22]. The inter-rater reliability in conventional MRI-based breast density estimations was moderate (*k* = 0.7, 95% CI 0.6, 0.79). Interestingly, the inter-rater reliability between mammographic and conventional MRI-based breast density readings revealed a minimal agreement (*k* = 0.3, 95% CI 0.17, 0.42). There was no difference in qualitative breast density between the craniocaudal view and left mediolateral-oblique view of the breast (*p* = .25).

**Table 1:** Patient characteristics

Parameter	Overall patient cohort *N* (%)
No. of patients	138
Age (median [IQR])	49.5 (43–56)
Breast characteristics	
ACR	
A	13 (9.4)
B	52 (37.7)
C	53 (38.4)
D	20 (14.5)
PDFF (median [IQR])	81.83 (73.31–87.97)
Breast cancer in history	
Yes	91 (65.9)
Surgery	58 (42.0)
Radiation therapy	40 (29)
Chemotherapy	28 (20.3)
Hormone therapy	24 (17.4)
No	47 (34.1)
Family history for breast cancer	
Positive	26 (18.8)
Negative	77 (55.8)
Gene mutation (including BRCA, FANCM, CHEK2)	
Yes	29 (21.0)
No	49 (35.5)

Patient and tumor characteristics. *IQR* interquartile range, *ACR* American College of Radiology categories, [A] almost entirely fatty, [B] scattered fibroglandular densities, [C] heterogeneously dense, and [D] extremely dense, *PDFF* proton density fat fraction, *BRCA* breast cancer gene, *FANCM* Fanconi anemia, complementation group M, *CHEK2* Checkpoint Kinase 2

**Fig. 3:**
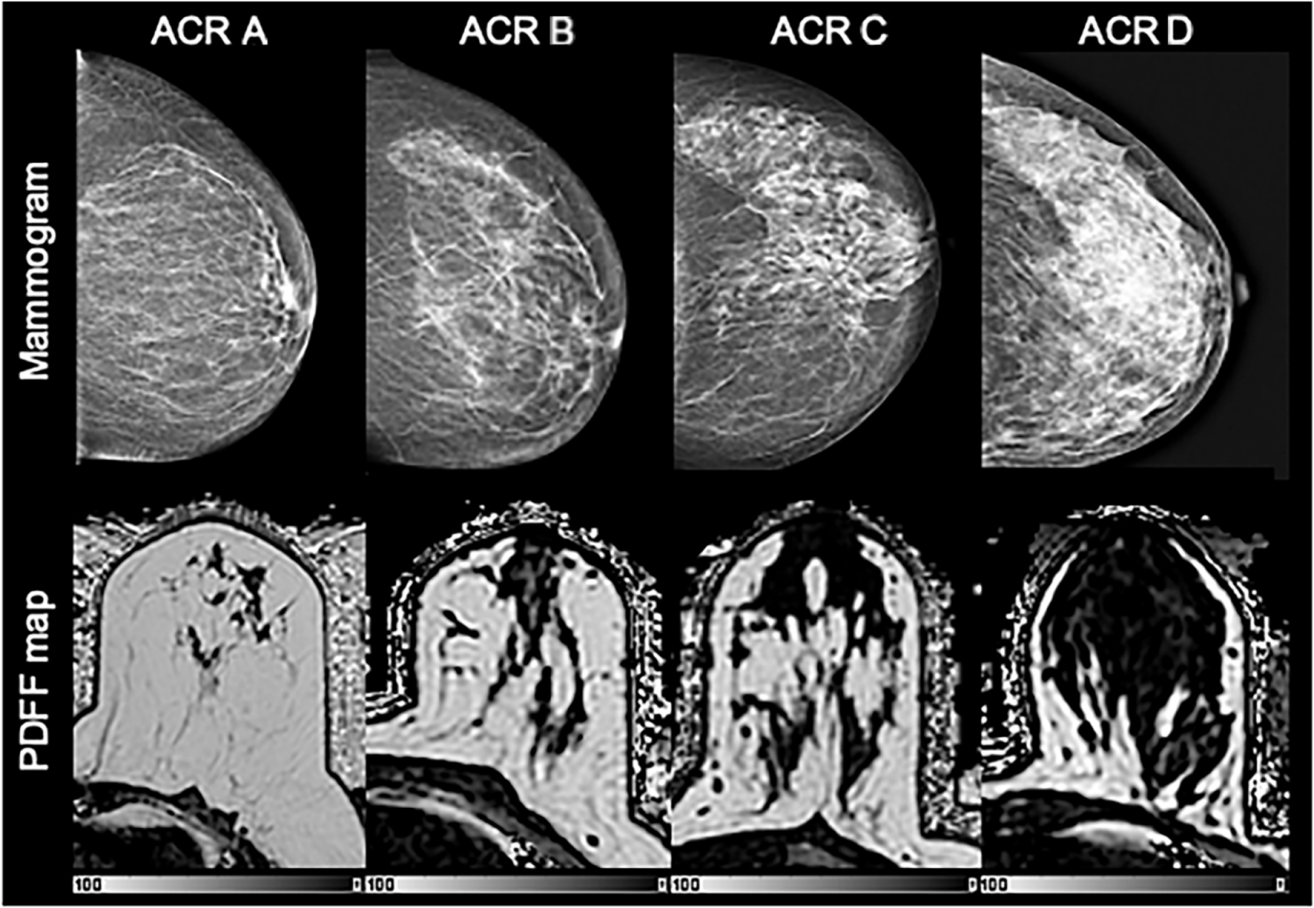
Mammograms with breast density estimation following the American College of Radiology Breast Imaging Reporting and Data System categories A-D with corresponding PDFF maps

**Fig. 4:**
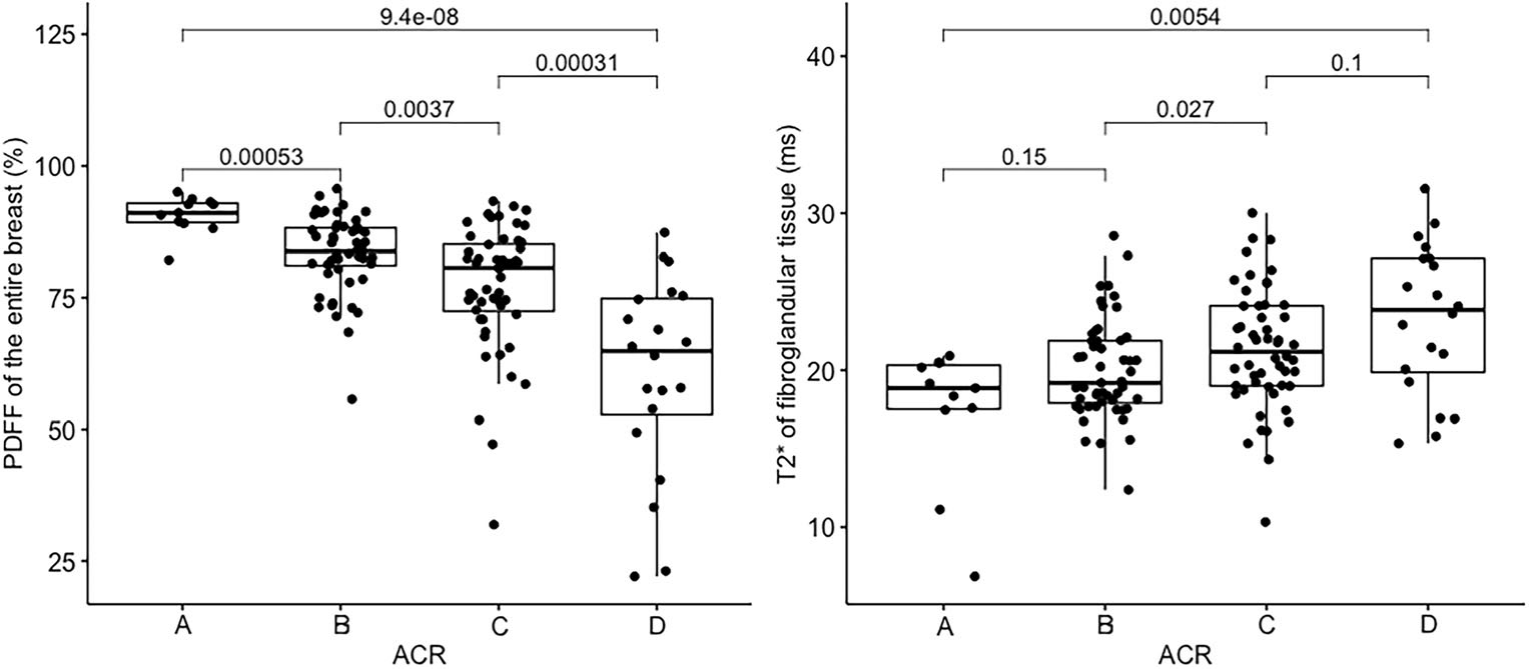
(A) Correlation of entire breast PDFF values to the four American College of Radiology Breast Imaging Reporting and Data System categories with adjusted *p* values revealing a significant negative correlation. (B) Significant correlation of T_2_* of fibroglandular tissue of the breast to ACR A-D

**Fig. 5:**
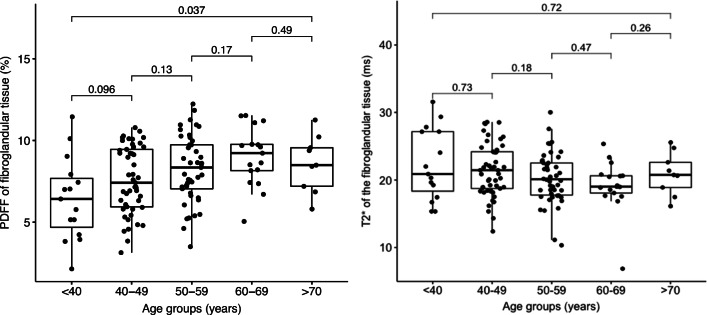
Correlation of mean PDFF values of the fibroglandular tissue to different age groups with adjusted *p* values revealing a positive correlation

Across all patients, the mean PDFF of the entire breast was 76.1 % and the mean PDFF of only the fibroglandular tissue was 7.7 %. Figure 3 shows an exemplary comparison of mammograms and PDFF maps of all four ACR categories. The PDFF of the entire breast strongly, negatively correlated with the mammographic breast density measurements (Spearman rho: −0.69, *p* < .001) and revealed a significant distinction between all four ACR categories following the Bonferroni-Holm correction (Fig. 4, Table 2). The PDFF of the entire breast also correlated with MRI-based visual estimation of the breast density (Spearman rho: −0.56, *p* < .001). Furthermore, the PDFF of fibroglandular tissue showed a correlation with age (logistic regression coefficient −0.02, *p* = .007, Pearson rho: 0.56, *p* = .03, Fig. 5). Mean T_2_* over the fibroglandular tissue also positively correlated with increasing ACR categories (Spearman rho: 0.34, *p* < .001). Mean T2* of the fibroglandular tissue did not correlate with age (*p* = .72, Fig. 5) but showed a trend towards shorter T2* values with increasing fat concentration stratified by ACR categories (*p* < .001).

**Table 2:** MRI parameter results stratified by the four American College of Radiology Breast Imaging Reporting and Data System categories (ACR A-D)

ACR	Mammography	Breast MR	PDFF entire breast	PDFF fibroglandular	T2* fibroglandular
	*N* (%)	*N* (%)	Median % (IQR)	Median % (IQR)	Median % (IQR)
ACR A	13 (9.4)	10 (7.2)	91.4 (89.8–93.2)	8.6 (7.6–9.5)	17.6 (15.2–20.7)
ACR B	52 (37.7)	52 (37.7)	85.1 (81.3–88.1)	8.5 (7.0–10.2)	19.9 (17.6–23.4)
ACR C	53 (38.4)	52 (37.7)	79.7 (73.8–84.8)	7.6 (6.1–9.3)	21.8 (19.8–24.4)
ACR D	20 (14.5)	24 (17.4)	64.0 (57.9–73.2)	5.8 (4.4–6.8)	23.1 (20.3–27.1)

MRI parameter results. *ACR* American College of Radiology according to the Breast Imaging Reporting and Data System (BI-RADS, 5th edition), [A] almost entirely fatty, [B] scattered fibroglandular densities, [C] heterogeneously dense, which may obscure small masses, and [D] extremely dense, *PDFF* proton density fat fraction, *IQR* interquartile range

## Discussion

The main finding of this study is that the breast PDFF strongly negatively correlates with mammographic and conventional MRI-based visual estimation of the breast density. The PDFF is an objective quantification of the breast density and in combination with T2* also provides information about the structural composition of the breast. The acquisition of the PDFF maps only requires a few minutes of MRI scan time which facilitates its applicability in clinical breast MR. Altogether, the PDFF allows for a robust, non-ionizing, quantitative alternative to measure the breast density and track structural alterations in the composition of the breast. Therefore, the PDFF can be used as a parameter for an individualized risk assessment for breast cancer.

Breast density estimation has emerged as a priority for assessing breast cancer risk as it is identified as one of the most prevalent risk factors for breast cancer and is implemented in most of the multifactorial risk assessment software tools [3, 23, 24]. Although radiographic mammography is the most widely used breast density measurement in clinical practice, its informative value is limited by a low intra- and inter-reader reliability. This discordance is influenced by the experience of the reporting radiologist and an incongruent technical execution [6, 25, 26]. Furthermore, the resulting two-dimensional projection of the volumetric body has a limited capacity to provide objective proportions of the breast composition, especially in dense breasts [4].

The clinical gold standard to estimate breast density remains mammography mainly due to its wide availability and relatively fast conductance. With the introduction of the BI-RADS classification system, the visual estimation of the breast density in T1-weighted MRI images was internationally standardized but is also still subject to moderate inter-rater reliability issues. The proposed, water-fat chemical-shift-based PDFF overcomes the 2D projection limitations of conventional mammography and presents a reliable, objective discriminator of tissue fat and water content. Previous works assessing breast density with MRI have relied on thresholding of T_1_-weighted and Dixon images [8, 10, 11, 27]. Tagliafico et al proposed a semi-automated method with an intensity threshold for dense breast parenchyma which is related to the entire breast volume [8]. Furthermore, Thomson et al used a defined threshold as a proposed MRI-based breast density measure calculated as the ratio of breast voxels with < 80% apparent fat fraction [27]. These approaches are susceptible to partial volume effects because of the sharp thresholding assigned to each breast voxel which ultimately increases breast density estimation errors. Besides, these methods did not account for the T_1_ bias which potentially results in a relative amplification of the signal of fat compared to the signal of water. The present work uses a low flip angle gradient echo sequence for mapping the PDFF, which is independent of the underlying relaxation properties and therefore reduces T_1_ bias. In another approach, Ding et al employed a Dixon acquisition using an IDEAL-GRASE sequence to quantify breast density based on the fraction of fibroglandular tissue and actual water content in each voxel. However, this sequence is limited by an intrinsic fat-water signal bias and therefore requires additional calibrations to remove T_1_- and T_2_-weighting effects [11]. In this study, a 6-echo gradient echo acquisition was used accounting for a single T_2_* decay and a pre-calibrated 7-peak fat spectrum accounting for the presence of multiple peaks in the fat spectrum in order to reduce potential confounding imaging factors, as also recently proposed [18]. In addition, scanning time remained under 3 min which renders the PDFF imaging sequence easily incorporable into clinical routine breast MRI exams.

Ascertained PDFF is independent of field strength, scanner platform, and specific scanning parameters and highly correlates with parenchymal triglyceride concentration [12]. Consequently, the PDFF has been already successfully applied in other organs and reliably quantified even small changes in tissue fat concentration [15, 28–30]. Unlike the signal fat-fraction, the PDFF reflects the actual content of fat in the breast and therefore presents a potentially reliable, standardized biomarker of breast density, as recently proposed [18]. In this study, the PDFF especially of the fibroglandular tissue also correlated with age which potentially mirrors the structural change of the breast with age [31]. Most prominent across the menopausal transition of 50–60 years, the fibroglandular tissue shows a natural decline in the amount of dense breast tissue with aging [32] that is also detectable with PDFF and T_2_* measurements. The observed dependence of fibroglandular tissue T_2_* with the ACR categorization might be also related to magnetic susceptibility differences between the water and fat components or to changes in the glandular component within the fibroglandular tissue [33]. The introduction of the PDFF (in combination with T2*) might provide additional and valuable information in the course of an individual risk assessment in screening patients and follow-up MRIs. The denser and more heterogeneously composed the breast presents, the higher is the risk to develop breast cancer but the lower is the detectability rate on mammography. As demonstrated in the DENSE trial, the additional use of MRI in clinical screening exams in very dense breasts with normal results in mammography significantly decreased the occurrence of interval cancers [34]. With the supplemental integration of the PDFF, the breast density can be objectively classified for a robust and standardized, individual risk assessment especially in patients with very dense and heterogeneously composed breasts to enable tailorized screening regimes. Furthermore, structural changes over time may be objectively tracked for an individualized risk assessment or treatment response estimation after cancer occurrence or anti-hormonal treatment [35, 36].

The present work has some limitations. First, it is focused on the relationship between breast PDFF and the mammographic and conventional MRI-based density metric. No validation of the reported fat fraction to another MR-based fat concentration measure was performed. However, the employed PDFF methodology has already accounted for confounding effects known from the application of the methodology in other organs. Second, no breast PDFF reproducibility analysis was presently performed. High reproducibility of PDFF for liver fat content and breast fat content in volunteers has been already confirmed [13, 37], therefore PDFF for breast parenchyma fat concentration in patients would be also expected to be high. Third, the employed methodology was based on a bipolar readout acquisition which accounted for phase errors in the quantification but showed failing behaviors in a small number of datasets. Further work using monopolar readout gradient could alleviate such problems [38].

The proposed breast PDFF mapping is a promising approach to an accurate, user-independent, and non-ionizing tissue fat concentration measurement that is directly comparable with visual mammographic, and conventional MRI-based breast density estimations. The improved concordance of the poor reader reproducibility, its platform and scan parameter independence, and the simple integration of the PDFF in clinical routine breast MRI exams may provide clinicians with a valuable tool for an individualized cancer risk assessment accurate and a reliable evaluation of longitudinal, structural changes in breast density and composition.

## Abbreviations

ACR: American College of Radiology
BI-RADS®: Breast Imaging Reporting and Data System
FOV: Field of view
ICC: Intraclass correlation coefficient
PDFF: Proton density fat fraction
SENSE: Sensitivity encoding

## Declarations

### Guarantor

The scientific guarantor of this publication is Prof. Dr. Dimitrios Karampinos from the Technical University Munich.

### Statistics and biometry

One of the authors (M.W.) has significant statistical expertise and gave statistical advice. However, no complex statistical methods were necessary for this paper.

### Methodology

• Retrospective

• Diagnostic or prognostic study

• Performed at one institution
